# Prevalence of celiac disease and celiac-related antibody status in pediatric patients with type 1 diabetes in Jordan

**DOI:** 10.1530/EC-19-0146

**Published:** 2019-05-14

**Authors:** Rasha Odeh, Abeer Alassaf, Lubna Gharaibeh, Sarah Ibrahim, Fareed Khdair Ahmad, Kamel Ajlouni

**Affiliations:** 1 Section of Pediatric Endocrinology, Department of Pediatrics, School of Medicine, University of Jordan, Amman, Jordan; 2 School of Pharmacy, University of Jordan, Amman, Jordan; 3 Section of Pediatric Gastroenterology, Department of Pediatrics, School of Medicine, University of Jordan, Amman, Jordan; 4 The National Center (Institute) for Diabetes, Endocrinology and Genetics (NCDEG), University of Jordan, Amman, Jordan

**Keywords:** type 1 diabetes, celiac disease, Jordan, prevalence, spontaneous normalization, predictors

## Abstract

**Objective:**

Scientific findings regarding the prevalence of celiac disease (CD) in pediatric patients with type 1 diabetes (T1D) in the Arab world are scarce. We aimed to determine the prevalence of biopsy-proven celiac disease (BPCD) among pediatric patients with T1D from Jordan. We also assessed the possible predictors for developing CD in this cohort of patients and we compared T1D patients who developed BPCD with those who had positive CD serology but negative histology and/or fluctuating CD serology.

**Methods:**

Celiac serology and duodenal biopsy results from 2012 to 2017 were collected from patients with T1D. The outcome of positive celiac serology and the risk factors for CD in T1D patients were investigated.

**Results:**

A total of 538 children of which 278 boys (51.7%) were included in the study. The prevalence of positive serology and the diagnosis of BPCD in this cohort of T1D patients were 16.6 and 9.1% respectively. Eighty percent of those with BPCD were asymptomatic and 47% were diagnosed with CD at onset of T1D. Spontaneous normalization of celiac serology occurred in 23.6% of those with positive serology.

**Conclusion:**

CD is prevalent in T1D pediatric patients from Jordan (9.1%). It is often asymptomatic and the majority of cases were diagnosed at onset or within 5 years of T1D diagnosis. Spontaneous normalization of CD serology occurred in some patients with T1D. Hence, a watchful follow-up is recommended in such patients.

## Introduction

Type 1 diabetes mellitus (T1D) is an immune-mediated disease that is known to be associated with other autoimmune conditions, most commonly thyroiditis and celiac disease (CD). While the prevalence of CD in the general population is estimated to be between 0.3 and 1% (1), it is significantly higher among patients with T1D due to a common genetic predisposition (2). The prevalence of CD ranges from 1 to 10% among children and adolescents with T1D (3, 4, 5, 6). A recent international comparative study with 53,000 children and adolescents with T1D across three continents reported a prevalence of CD of 3.5%, with rates ranging from 1.9% in the United States to 7.7% in Australia (7).

There are limited data on the prevalence of CD in Arab children with T1D. The prevalence in the reported studies from Saudi Arabia, Tunisia, West Algeria, Libya and Oman ranges between 4.9 and 11.3% (8, 9, 10, 11, 12, 13, 14). There are no data about the prevalence of biopsy-proven CD (BPCD) among children and adolescents with T1D from Jordan.

Due to this high prevalence of CD among pediatric patients with T1D, the potential clinical consequences and the fact that it is often asymptomatic, screening for positive CD serology is recommended for all patients with T1D soon after diagnosis (15, 16, 17, 18). Also, it is recommended that screening should be repeated at 2- and 5-year intervals or sooner if the patient is symptomatic or has a first-degree relative with CD, as it has been shown that positive celiac serology conversion is highest in the first 5–10 years after T1D diagnosis (3, 5, 17, 18). Recent studies have also shown a significant spontaneous normalization of celiac serology in patients with T1D despite continuing ingestion of a gluten-containing diet (19).

In this study we aim to determine, for the first time, the prevalence of BPCD among pediatric patients with T1D from Jordan by describing the development of celiac antibodies and BPCD in these patients, paying specific attention to timing of positive antibodies, disease development and spontaneous normalization of antibody titers. We also assess the possible predictors for developing CD in this cohort of patients and we compare T1D patients who developed BPCD with those who had positive CD serology but negative histology and or fluctuating CD serology.

## Methods and patients

This is a mixed prospective and retrospective cohort study, based on review of medical files and electronic medical records and follow-up. All T1D patients attending the pediatric endocrinology clinics at the Jordan University Hospital (JUH) and the National Center for Diabetes Endocrinology and Genetics (NCDEG) (two major referral centers for T1D in Amman, Jordan) between 2012 and 2017 were included. Since the establishment of the pediatric diabetes clinics in these two centers in 2012, all T1D patients were screened for CD-related antibodies at onset of their disease and annually. In addition, all patients who presented at a later stage of their T1D were asked to provide any previous CD screening results. Those who were not previously screened underwent CD antibody screening. In the presence of a high CD antibody titer or clinical symptoms (whether gastrointestinal or extra-gastrointestinal), children were offered endoscopy for the histological diagnosis. Asymptomatic patients showing low CD antibody titers were invited to have a second serological determination after 4–6 months of following a gluten-containing diet and if still positive, they were offered endoscopy. Data collected for each patient included: date of birth, sex, date of T1D diagnosis, dates and results of CD serology tests, dates and results of duodenal biopsies, date of CD diagnosis, associated thyroid disease, family history of autoimmune diseases and symptoms of CD in patients with positive serology.

### CD-related antibody determinations

In JUH, CD serology testing was determined by total serum IgA and anti-tissue transglutaminase IgA (tTG IgA) using a commercially obtained ELISA (enzyme linked immunosorbent assay) kit, anti-huTransG (Generic Assays, Dahlewitz, Germany). In NCDEG, serology was determined by tTG IgA and tTG IgG using the same method. An ELISA cut-off value of less than 20 IU/mL was considered normal and equal or greater than 20 IU/mL, positive according to the manufacturer’s instructions. Before 2015, NCDEG used another commercial ELISA kit for which the cut-off level was 15 IU/mL. Serological autoantibody titers were recorded as multiplications of the upper limit of normal (ULN) to adjust for this cut-off difference.

### Histological examination of duodenal biopsies

The majority of patients who were eligible for endoscopy underwent the procedure at JUH where at least four biopsy specimens in each patient were obtained including the duodenal cap. A pathologist interpreted the samples according to the histological criteria described in the Marsh classification (20). However, a minority of patients underwent the endoscopy in other hospitals and reports of the results were gathered. BPCD was diagnosed if the patient had Marsh ≥3 score in the duodenal biopsy. The last patient enrolled in the current study was in 2017 in order to have a follow-up of at least 1 year. Finally, the cohort comprised patients with T1D only and those with both T1D and CD. Those who had positive serum antibody serologies but negative biopsies or were followed without biopsies were designated as having a fluctuating serum serology. The study was approved by the Institutional Ethics Committees in both JUH and NCDEG.

### Statistical analysis

Continuous data are presented as mean ± s.d., and categorical data as frequency (%). Associations between categorical dependent variables and independent categorical variables were evaluated using chi-squared analysis. Possible predictors of CD were assessed using logistic regression (Entre method) analysis. Associations between categorical dependent variables and independent continuous variables were evaluated using independent *t*-test. *P* values less than 0.05 were considered statistically significant.

## Results

### Patients with type 1 diabetes

**Figure 1:**
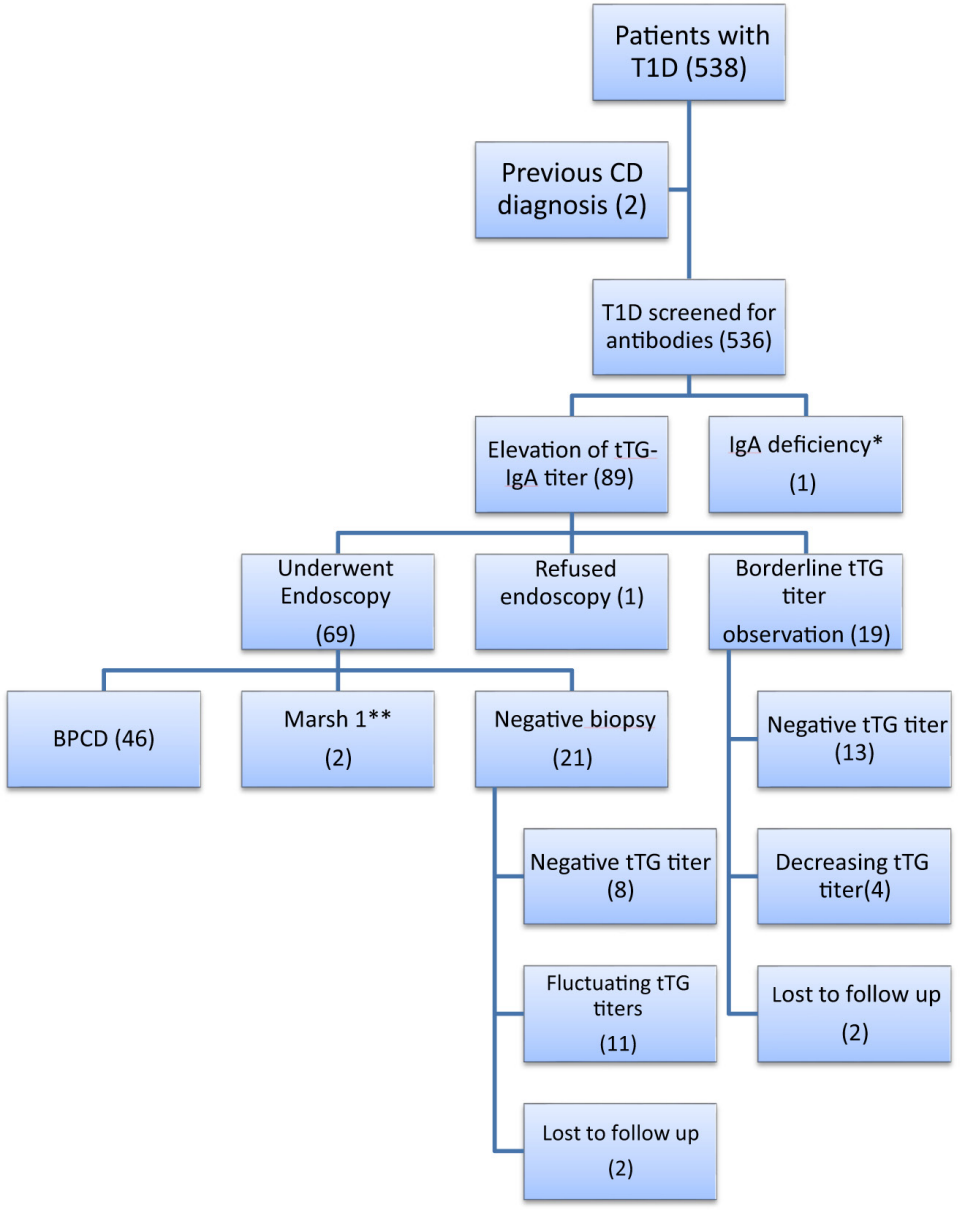
Study participants flow chart. *This patient was found to have BPCD (Marsh ≥3). **These two patients had infiltrative disease (Marsh 1) and were added to the group of patients with T1D and fluctuating CD serology. BPCD, biopsy-proven celiac disease; CD, celiac disease; T1D, type 1 diabetes; tTG IgA, anti-tissue transglutaminase immunoglobulin A.

A total of 538 children (278 boys (51.7%)) were included in the study. The mean age of patients was 12.02 ± 3.94 years and the mean duration of T1D at the time of the study was 4.43 ± 2.65 years. The mean age at T1D diagnosis was 7.56 ± 3.64 years. Two children had a diagnosis of CD before T1D onset. The evolution of the cohort is shown in Fig. 1.

### Diagnosis of CD in T1D patients

After excluding the two patients with the diagnosis of CD before the onset of T1D, the prevalence of patients with positive celiac serology at the end of the study period was 89/536 (16.6%). One patient had total serum IgA deficiency with gastrointestinal symptoms; at onset of her T1D and was proven to have CD by histology. Another patient refused endoscopy and was clinically considered to have CD due to persistence of high tTG IgA titer (>15 ULN) and the presence of gastrointestinal symptoms. Out of the 89 patients with positive serology for CD, 46 (51.7%) were diagnosed with BPCD. An additional 19 subjects did not undergo endoscopy and were followed clinically due to borderline tTG IgA titers and absence of symptoms (Fig. 1).

### Histology results

Out of a total of 70 patients who underwent duodenal biopsy, Marsh ≥3 changes (various degrees of villous atrophy with crypt hyperplasia and increased intraepithelial lymphocytosis) were present in 47 patients. An additional two patients had intraepithelial lymphocytosis with preserved villous architecture (Marsh 1). The remaining patients (21/70) showed negative histological features of CD. Of all participants, the proportion of patients with a final diagnosis of BPCD (Marsh ≥3) was 49/538 (9.1%).

### Spontaneous normalization of CD serology

During follow up, 21 out of the 89 subjects who had positive CD serology became tTG IgA negative. Of these, eight underwent endoscopy and had negative results. Fifteen subjects (16.9%) continued to show fluctuating titers of tTG IgA. The percentage of patients who had positive CD serology and then showed spontaneous normalization of CD antibody titers in our cohort was 23.6%. Subjects with Marsh 1 histopathological results and subjects with spontaneous normalization of CD serology, in addition to the ones with negative biopsies and/or persistent positive CD serology constitute the group of T1D with fluctuating CD serology (42/89 (47.2%)).

### Time from onset of T1D to final diagnosis of CD

Out of the 49 patients that were diagnosed with BPCD, 23 subjects (47%) were diagnosed with CD at the onset of their T1D diagnosis as a result of antibody screening. After excluding patients with BPCD diagnosed before and at onset of diabetes, 18/24 (75%) and 21/24 (87.5%) of patients had CD diagnosed by 3 and 5 years after onset of T1D, respectively (Table 1). In addition, 2 out of the 3 patients who were diagnosed with CD after 5 years of T1D onset had no previous documented serological screening for CD. One of them had chronic constipation and the other had short stature with no gastrointestinal symptoms.

**Table 1:** Intervals between T1D diagnosis and BPCD diagnosis (*N* = 49).

Interval	Before T1D Dx	At T1D Dx	1st year	2nd year	3rd year	4th year	5th year	≥6th year
No. of cases (%)	2 (4.1)	23 (47)	8 (16.3)	5 (10.2)	5 (10.2)	1 (2)	2 (4.1)	3 (6.1)

This table shows the numbers of cases diagnosed with BPCD in relation to the timing of T1D diagnosis.

BPCD, biopsy-proven celiac disease; Dx, diagnosis; T1D, type 1 diabetes.

### Symptoms and signs of CD in patients with T1D and CD

In patients having CD at onset or after T1D diagnosis, 20% displayed symptoms of CD, with 15.7% having gastrointestinal symptoms and 4.3% having non-gastrointestinal symptoms (short stature and recurrent unexplained hypoglycemia).

### Comparison between patients with T1D only and patients with T1D and CD

The characteristics of T1D patients with and without CD are shown in Table 2. Mean age at T1D diagnosis was not statistically significantly different between patients with T1D only compared to those diagnosed with both T1D and CD (7.67 ± 3.65 vs 6.61 ± 3.43 years, *P* = 0.057). In addition, there was no significant difference in the current age between the two groups (12.04 ± 3.93 vs 11.90 ± 3.99 years, *P* = 0.816). However, the duration of diabetes was significantly shorter in patients with T1D only compared to those diagnosed with both T1D and CD (4.35 ± 2.58 vs 5.31 ± 3.18 years, *P* = 0.046). Different variables were assessed as possible predictors of CD. Two variables showed statistical significance (Table 3). In multivariate analysis, each one-fold increase in the tTG IgA above ULN increased the likelihood of having CD by 22%. In the univariate analysis, children who were diagnosed with T1D aged between 5 and 10 years had a 52% less likelihood of having CD compared to those who were diagnosed at a younger age (less than 5 years).

**Table 2:** Characteristics of T1D patients with and without CD (*N* = 536).

	T1D only (*N* = 488, 91%)	T1D+CD (*N* = 48, 9%)	P* value
Female	236 (48.4)	26 (54.2)	0.443
Presence of hypothyroidism	18 (3.7)	4 (8.3)	0.122
Family history of autoimmune disease	173 (35.5)	22 (45.8)	0.154
Age at diagnosis of T1D (years)			0.062
<5	126 (25.8)	20 (41.7)	
5–10	263 (53.9)	20 (41.7)	
>10	99 (20.3)	8 (16.7)	

*Using chi-square.

CD, celiac disease; T1D, type 1 diabetes.

**Table 3:** Possible predictors of celiac disease in type 1 diabetes patients.

Variables	Univariate analysis			Multivariate analysis		
	OR	95% CI	P	OR	95% CI	P*
Gender						
Male^α^						
Female	1.26	0.70–2.29	0.443	0.98	0.36–2.68	0.971
Presence of hypothyroid disease						
No^α^						
Yes	2.37	0.77–7.32	0.133	1.51	0.21–10.67	0.680
tTG IgA (ULN)^∞^	1.23	1.12–1.35	<0.001	1.22	1.11–1.34	<0.001
Age at T1D diagnosis (years)						
<5^α^						
5–10	0.48	0.25–0.92	0.028	0.470	0.15–1.48	0.198
>10	0.51	0.22–1.20	0.124	0.440	0.11–1.71	0.236

^α^Reference; **^∞^**Folds more than the upper normal level of tissue transglutaminase IgA; *Using logistic regression.

We also evaluated the differences between the characteristics of children with T1D and CD compared to those with fluctuating celiac serology (Table 4). Both the age at diabetes diagnosis and the duration of diabetes were significantly different between the two groups. Children with CD were diagnosed with diabetes at a younger age and had a longer duration of diabetes than those with fluctuating celiac serology. Twenty-six of children (54%) with BPCD were females, compared to 21 (50%) of the group with fluctuating celiac serology. This difference was not statistically significant (*P* = 0.693).

**Table 4:** Characteristics of patients with T1D and celiac disease and those with fluctuating celiac serology (*N* = 90).

Variables	T1D with celiac disease (*N* = 48)	T1D with fluctuating celiac serology (*N* = 42)	P*
	Mean ± SD	Mean ± SD	
Age at T1D diagnosis (years)	6.61 ± 3.43	8.40 ± 3.41	0.015
Duration of T1D (years)	5.31 ± 3.18	3.79 ± 1.80	0.006
Current age (years)	11.90 ± 3.99	12.26 ± 3.36	0.654

*Using independent *t*-test.

T1D, type 1 diabetes.

## Discussion

The prevalence of positive tTG IgA serology and BPCD in our cohort of T1D patients was 16.6 and 9.1% respectively. The prevalence of positive CD serology in the general pediatric population in Jordan is estimated to be 1.5% using tTG IgA and 0.8% using both tTg IgA and anti endomysial IgA antibodies (21). This confirms that pediatric patients with T1D from Jordan have almost a 10 times higher risk of developing positive CD serology than the general population. In addition, Albatyneh *et al*. estimated the prevalence of positive tTG IgA in 138 patients with T1D from south Jordan to be 6.5% (22). Our pediatric cohort showed more than twice that number, which could be due to a larger patient cohort and the fact that our centers cover referrals from all over the country. To our knowledge, this is the first report about the prevalence of BPCD in pediatric patients with T1D from Jordan. Our findings still lie within the reported prevalences worldwide (1–10%) and in Arab countries (4.9–11.3%), though at the upper end of this range (3, 4, 5, 6, 7, 8, 9, 10, 11, 12, 13, 14). This relatively high prevalence is probably due to a predisposing genetic susceptibility in our population. This needs further investigation.

The timing of CD diagnosis in our patients was mostly at onset and within 3–5 years of their T1D diagnosis. This finding is similar to other cohorts from different parts of the world namely, Sweden, Australia and Italy (3, 5, 23). However, no data are available from the Arab world to describe the relationships between both; the onset of positive CD serology and BPCD diagnosis, and the time of T1D diagnosis as reports from the Arab world were cross-sectional reporting prevalence only (8, 9, 10, 11, 12, 13, 14). Hence, our findings suggest that the international guidelines proposing CD screening for patients with new onset T1D and close follow-up in the first 5 years (17, 18) also apply to our geographical region.

The strong association between T1D and CD is associated with a common genetic susceptibility attributed to specific shared alleles, with HLA-DR3-DQ2 and DR4-DQ8 haplotypes increasing the risk of both diseases (24, 25). Interestingly, it has been shown that children with HLA-conferred susceptibility to T1D and CD develop CD-associated antibodies mostly at a younger age or at the same age at which they develop diabetes-associated auto-antibodies (26). However, clinical T1D is usually diagnosed before CD. In fact, CD diagnosis often occurs from screening at onset of the diabetes and during follow-up (5, 23). Some authors hypothesized that in genetically susceptible patients; untreated (latent or silent) CD could be an immunological trigger and induce diabetes due to gluten as a driving antigen. However, this has not been confirmed as reports show no correlation between the duration of gluten exposure in adult CD and the risk of autoimmune disorders (31, 32). Hence, gluten is currently considered as a modifier rather than a determinant of the pathogenesis of T1D, facilitating the progression of other dietary factors to the lamina propria, to activate the autoimmune response against beta cells (27).

Some groups have investigated the association between the occurrence of CD and the age at T1D diagnosis. Two large pediatric studies from Australia and Italy have shown that diagnosis of T1D at a younger age (<5 years) increased the risk of developing CD, after a longer diabetes duration, than when T1D presented at an older age (5, 27). However, this was not a consistent finding, as other reports found no difference (3, 28, 29). In our cohort, there was a tendency for younger patients at T1D onset to have higher risk of developing CD but the difference was marginal.

In addition, several studies reported a difference in CD prevalence in T1D patients with gender; the prevalence being higher in girls in two studies (27, 33), higher in boys in three studies (3, 29, 34) and not different in two further studies (5, 28). We did not find any difference in gender susceptibility.

The classical clinical features of CD include gastrointestinal and extra-gastrointestinal manifestations such as chronic or intermittent diarrhea and/or constipation, chronic abdominal pain/distention, flatulence, anorexia, dyspeptic symptoms as well as anemia, vitamin deficiencies, poor linear growth, delayed puberty or recurrent aphthous ulceration (35, 36). These symptoms and deterioration in glycemic control or hypoglycemia should alert the health care professional to investigate and exclude CD. However, many pediatric T1D patients have asymptomatic CD and the diagnosis was made as a result of CD antibody screening (15, 16) as was the case in our patients. Current recommendations advise that children with CD should start a gluten-free diet under the supervision of an experienced pediatric dietitian. Barriers to adherence to such a diet include the burden of a second disease, high cost and unavailability of gluten-free products, problems with sensory acceptance, inadequate support from family and peers, absence of symptoms following ingestion of gluten, and lack of knowledge of the health-related complications of gluten ingestion (16).

The recommendation of a small bowel biopsy for all T1D patients with positive CD serology has been recently challenged by several investigators who demonstrated spontaneous normalization of CD serology over varying periods of time in 20–35% of cases (19, 37). The presence of CD symptoms and younger age at onset of T1D were the strongest predictors of positive CD serology, also endomyseal antibody positivity and a tTG IgA antibody level greater than the seven- to eightfold the ULN value were strong predictors of BPCD diagnosis (19). We observed spontaneous normalization of CD serology in approximately a quarter of the patients who initially tested positive. The strongest predictor for BPCD was the high tTG IgA titer levels.

In conclusion, CD is prevalent in T1D patients from Jordan (9.1%). It is often asymptomatic and around half the cases were diagnosed as a result of screening at onset of T1D. After that, CD is diagnosed mostly within the first 5 years after T1D diagnosis. Spontaneous normalization of CD serology was observed in 23.6% of patients who mostly had borderline elevations of tTG IgA with no symptoms. Hence, a watchful follow-up is recommended in such patients.

## Declaration of interest

The authors declare that there is no conflict of interest that could be perceived as prejudicing the impartiality of the research reported.

## Funding

This work was supported by a grant from the deanship of academic research, University of Jordan (grant number 138/2016-2017).
